# Reduced volume of the left cerebellar lobule VIIb and its increased connectivity within the cerebellum predict more general psychopathology one year later via worse cognitive flexibility in children

**DOI:** 10.1016/j.dcn.2023.101296

**Published:** 2023-09-07

**Authors:** Gai Zhao, Haibo Zhang, Leilei Ma, Yanpei Wang, Rui Chen, Ningyu Liu, Weiwei Men, Shuping Tan, Jia-Hong Gao, Shaozheng Qin, Yong He, Qi Dong, Sha Tao

**Affiliations:** a State Key Laboratory of Cognitive Neuroscience and Learning, Beijing Normal University, Beijing 100875, China; b IDG/McGovern Institute for Brain Research, Beijing Normal University, Beijing 100875, China; c Center for MRI Research, Academy for Advanced Interdisciplinary Studies, Peking University, Beijing 100871, China; d Psychiatry Research Center, Beijing Huilongguan Hospital, Peking University, Beijing 100096, China

**Keywords:** P factor, Left cerebellar lobule VIIb, Connectivity, Cognitive flexibility, School-age children

## Abstract

Predicting the risk for general psychopathology (the p factor) requires the examination of multiple factors ranging from brain to cognitive skills. While an increasing number of findings have reported the roles of the cerebral cortex and executive functions, it is much less clear whether and how the cerebellum and cognitive flexibility (a core component of executive function) may be associated with the risk for general psychopathology. Based on the data from more than 400 children aged 6–12 in the Children School Functions and Brain Development (CBD) Project, this study examined whether the left cerebellar lobule VIIb and its connectivity within the cerebellum may prospectively predict the risk for general psychopathology one year later and whether cognitive flexibility may mediate such predictions in school-age children. The reduced gray matter volume in the left cerebellar lobule VIIb and the increased connectivity of this region to the left cerebellar lobule VI prospectively predicted the risk for general psychopathology and was partially mediated by worse cognitive flexibility. Deficits in cognitive flexibility may play an important role in linking cerebellar structure and function to the risk for general psychopathology.

## 1 Introduction

The risk for general psychopathology (the p factor) has drawn increasing attention since it may account for the shared risk of both internalizing and externalizing problems in school-age children (Brislin et al., 2022, Lahey et al., 2015, Patalay et al., 2015, Snyder et al., 2017). For school-age children, a higher p factor suggests a relatively higher risk for developing a future clinical disorder (Caspi and Moffitt, 2018). While a few studies have shown cerebral correlates for the risk for general psychopathology in children and adolescents (Kaczkurkin et al., 2019, Snyder et al., 2017), whether and how features of the cerebellum may predict the risk for general psychopathology remains largely unknown. Here, we aimed to examine whether and how the cerebellum influences the development of the risk for general psychopathology in school-age children for the first time.

The human cerebellum represents almost 80% of the surface area of the neocortex (Sereno et al., 2020) and is involved in various psychiatric disorders in children and adolescents, such as depression (Chang et al., 2018, Gaffrey et al., 2012), anxiety disorder (Burkhouse et al., 2019, Lange et al., 2015), autism spectrum disorder (ASD) (Bednarz and Kana, 2019, Guo et al., 2021, Khan et al., 2015), and attention-deficit/hyperactivity disorder (ADHD) (Bledsoe et al., 2011, Castellanos et al., 2002, Lantieri et al., 2010, Mackie et al., 2007, Shaw et al., 2018). It is plausible that the structural and functional connectivity of the cerebellum may also be associated with the risk for general psychopathology in children and adolescents. Several cross-sectional studies found that reduced posterior cerebellar gray matter, especially of the left cerebellar lobule VIIb, is associated with a higher risk for general psychopathology in children and adolescents (Moberget et al., 2019) and college students (Romer et al., 2018). However, previous cross-sectional studies were unable to reveal whether the gray matter volume of the left cerebellar lobule VIIb and its connection within the cerebellum may precede the development of the risk for general psychopathology. Moreover, the development of cognitive flexibility in children and adolescents is closely associated with the left cerebellar lobule VIIb (Lie et al., 2006, Moore et al., 2017) and the risk for general psychopathology (Bloemen et al., 2018, Caspi et al., 2014). It is plausible that cognitive flexibility may mediate the longitudinal associations between the gray matter volume of the left cerebellar lobule VIIb, its connectivity within the cerebellum, and the risk for general psychopathology. Thus, this study aimed to examine the longitudinal relationships between the left cerebellar lobule VIIb, its connectivity within the cerebellum and the risk for general psychopathology, as well as the mediating role of cognitive flexibility in these longitudinal relationships in school-age children.

The left cerebellar lobule VIIb has been found to be associated with executive function (Buckner et al., 2011, Moore et al., 2017), and deficits in executive function are associated with a higher risk for general psychopathology in children and adolescents (Harden et al., 2020, Martel et al., 2017, Romer and Pizzagalli, 2021, Shields et al., 2019, Snyder et al., 2019, Wade et al., 2020). Abnormalities of the left cerebellar lobule VIIb may play a critical role in the development of a higher risk for general psychopathology in children and adolescents. For example, a cross-sectional study of children and adolescents aged 8–23 showed that cerebellar posterior gray matter volume encompassing bilateral lobules VI, Crus I, VIIb, and VIIIa predicted the risk for general psychopathology much better than other cerebral cortex anatomical features (Moberget et al., 2019). Another cross-sectional study of college students revealed that reduced gray matter volume in the left cerebellar lobule VIIb was associated with a higher the risk for general psychopathology (Romer et al., 2018). However, two recent studies failed to replicate the link between the gray matter volume of the left cerebellar lobule VIIb and the risk for general psychopathology in middle-aged adults (Romer et al., 2021, Romer and Pizzagalli, 2022). And neither study identified other cerebellar regions that were significantly associated with the risk for general psychopathology. Therefore, age differences in samples from previous studies may lead to inconsistent conclusions. Taken together, the findings of these studies may suggest that the left cerebellar lobule VIIb is particularly important in determining the risk for general psychopathology in children and adolescents. Given that the inclusion of subjects with a broad age range (e.g., 8–23 years) may obscure developmental changes in these brain-behavior relationships (Durham et al., 2021), the associations between the left cerebellar lobule VIIb and the risk for general psychopathology could be mapped more precisely in a sample with a more homogeneous age range (e.g., school-age children).

A reduced gray matter volume of the left cerebellar lobule VIIb may play a critical role in the higher risk for general psychopathology (Romer et al., 2018). Previous studies have demonstrated that the morphological structure constrains functional connectivity (Pang et al., 2023; Schutter, 2021; Segall et al., 2012; Yee et al., 2018); therefore, we speculated that the functional connectivity of the left cerebellar lobule VIIb within the cerebellum may be related to the p factor. More importantly, previous studies have demonstrated that intra-cerebellar functional connectivity is associated with diverse mental disorders, such as schizophrenia, cognitive affective syndrome, and posttraumatic stress and anxiety disorder (Dong et al., 2022, Feng et al., 2022, Gil-Paterna and Furmark, 2023, Stoodley and Schmahmann, 2023). The intra-cerebellar functional connectivity may be related to the risk for general psychopathology. Altogether, exploring the structural features of left cerebellar lobule VIIb and its functional connectivity within the cerebellum could provide valuable insights into the relationship between the risk for general psychopathology and the cerebellum. Moreover, previous studies have shown that a higher risk for general psychopathology is significantly linked to increased functional connectivity within the brain (Elliott et al., 2018, Lees et al., 2021). Based on this, the increased functional connections within the cerebellum may also be significantly associated with a higher risk for general psychopathology. Furthermore, previous well-documented evidence supports that neuroimaging features of psychiatric problems precede the development of psychiatric problems in children and adolescents (Albaugh et al., 2019, Ing et al., 2019, Shaw et al., 2006, Whelan et al., 2014). These cross-sectional studies have shown that reduced gray matter volume of the left cerebellar lobule VIIb is significantly associated with a high risk for psychopathology; however, little is known about this association and how its connection to the cerebellum precedes the development of the risk for general psychopathology. Thus, we collected longitudinal data to examine whether the left cerebellar lobule VIIb and its regulation of the cerebellum prospectively predict the risk for general psychopathology in school-age children.

Despite findings from several cross-sectional studies on the relationship between the left cerebellar lobule VIIb and the risk for general psychopathology, little is known about how the left cerebellar lobule VIIb and its connectivity within the cerebellum may affect the development of the risk for general psychopathology over time. Cognitive flexibility, a core component of executive function (Diamond, 2013, Miyake et al., 2000), may play a mediating role in the links between the left cerebellar lobule VIIb and the risk for general psychopathology. On the one hand, the development of cognitive flexibility may be supported by the left cerebellar lobule VIIb in children and adolescents (Moore et al., 2017) and adults (Lie et al., 2006). On the other hand, cognitive flexibility may be associated with the risk for general psychopathology. The impaired disengagement hypothesis (Koster et al., 2011) proposed that cognitive flexibility deficits hinder the ability to perceive challenging situations from multiple perspectives, leading to negative emotions and an increased risk for general psychopathology. Cross-sectional studies have revealed a negative correlation between cognitive flexibility and the risk for general psychopathology (Bloemen et al., 2018, Caspi et al., 2014). Recently, a longitudinal study found that worse cognitive flexibility predicted a higher risk for general psychopathology one year later in Chinese school-age children (Zhao et al., in press). Based on these findings, it is plausible to suggest that cognitive flexibility may mediate the longitudinal associations between the left cerebellar lobule VIIb, its connectivity within the cerebellum, and the risk for general psychopathology.

This study aimed to examine whether the left cerebellar lobule VIIb and its connectivity within the cerebellum may prospectively predict the risk for general psychopathology and whether cognitive flexibility may mediate the predictive relationships in school-age children. First, we examined whether the gray matter volume of the left cerebellar lobule VIIb and its modulation of the cerebellum prospectively predicted the risk for general psychopathology among school-age children. We hypothesized that a smaller gray matter volume of the left cerebellar lobule VIIb and its increased connectivity within the cerebellum preceded the development of the risk for general psychopathology among school-age children. Second, we examined whether cognitive flexibility may mediate the longitudinal associations between the gray matter volume of the left cerebellar lobule VIIb, its connectivity within the cerebellum and the risk for general psychopathology among school-age children. We hypothesized that the smaller gray matter volume of the left cerebellar lobule VIIb and its increased connectivity within the cerebellum may increase the risk for general psychopathology (higher p factor) via worse cognitive flexibility among school-age children.

## 2 Methods

### 2.1 Participants

Neuroimaging and behavioral data were obtained from the Children School Functions and Brain Development Project (CBD, Beijing Cohort). CBD is a large ongoing accelerated longitudinal cohort of school-age children who have undergone comprehensive assessments including annual multimodal MRI scans of the brain, assessments of physical health, cognitive and noncognitive functions, and academic achievement. In the present study, the data were collected at two time points with approximately one-year intervals (10–16 months, average 12.187 months). Participants recruited in this study were cognitively normal and had no history of neuropsychiatric illness, psychoactive drug use, significant head injuries, or significant physical illness. Informed consent was obtained from the parents/guardians (written) and children (oral). All study procedures were reviewed and approved by the Institutional Review Board at Beijing Normal University in accordance with the Declaration of Helsinki.

At baseline, 508 typically developing children completed the T1 MRI data collection. Among them, 473 children met the quality criteria for T1 MRI data and had complete data on cognitive flexibility and psychopathology. However, at the one-year follow-up, 220 children did not have complete psychopathology data. Thus, the final sample consisted of 253 children with T1 MRI and cognitive flexibility data at baseline, as well as psychopathology data at both baseline and follow-up (48.20% girls, mean age = 9.129 ± 1.487 years at baseline, mean age = 10.145 ± 1.521 years at follow-up). Regarding resting-state fMRI data, at baseline, 447 typically developing children completed the data collection. Among them, 439 children met the quality criteria for resting-state fMRI data, and they all had complete data on cognitive flexibility and psychopathology; at the one-year follow-up, 209 children did not have complete data on psychopathology. Thus, the final sample consisted of 230 children with resting-state fMRI and cognitive flexibility data at baseline, as well as psychopathology data at both baseline and follow-up (48.30% girls, mean age = 9.152 ± 1.464 years at baseline, mean age = 10.176 ± 1.492 years at follow-up).

Since CBD is a large, ongoing accelerated longitudinal study and follow-up data are currently being collected, some data were not yet collected when we performed the analysis. The issue here is not whether there is less follow-up data, but whether the data from certain participants at baseline but not follow-up influence the results. To this end, we investigated these data patterns by testing whether demographics may be associated with differential attrition in the data by referring to previous studies (Wang and Liu, 2021, Zhao et al., 2023). The children with psychopathology data at baseline only and not follow-up (*n* = 220) and the children included in our structural MRI analytic sample (*n*_Structural MRI Study_ = 253) were not different in terms of gender (χ^2^_(1)_ = 2.873, *p* = 0.090), site (χ^2^_(1)_ = 0.074, *p* = 0.786), age (*t*_(471)_ = −0.762, *p* = 0.446) and parental education level (*t*_(471)_ = 0.376, *p* = 0.707). Similarly, the children with psychopathology data at baseline only and not follow-up (*n* = 209) and children included in our resting state fMRI analytic sample (*n*_Resting-state fMRI Study_ = 230) were not different in terms of gender (χ^2^_(1)_ = 1.674, *p* = 0.196), site (χ^2^_(1)_ = 1.792, *p* = 0.181), age (*t*_(437)_ = −1.013, *p* = 0.312) or parental education level (*t*_(437)_ = 1.108, *p* = 0.281). In conclusion, the patterns of participants with data at baseline only and not follow-up were not different from the patterns of those included in our analysis.

### 2.2 Psychopathology assessment

The parent-reported version of the Strengths and Difficulties Questionnaire (SDQ) was used to assess children’s psychopathology, including internalizing and externalizing problems (Goodman, 1997). Parents completed the Chinese version of the SDQ at baseline and follow-up in our study. The Cronbach’s α for the baseline and follow-up were 0.78 and 0.79, respectively. Previous studies have shown that the parent-reported SDQ has high levels of reliability and validity, indicating that it is appropriate for assessing psychopathology in Chinese children and adolescents (Du et al., 2008, Lai et al., 2014, Liu et al., 2013).

### 2.3 Cognitive flexibility assessment

The Wisconsin Card Sorting Test (WCST) has been used to assess cognitive flexibility (Grant and Berg, 1948) in a wide age range (6–89 years) and involves four target cards and 128 response cards. Each card shows different forms (crosses, circles, triangles, or stars), colors (green, yellow, blue or red), and numbers of figures (one, two, three or four figures). The task was to match an aspect of each card (color, shape, or number) from the deck of 128 cards that were provided to the participant to the target cards, one by one. Participants clicked on one of the four cards at the top of the screen that they thought matched the card at the bottom of the screen. Although the rules for sorting the cards were not explicitly stated, participants were informed by the computer after each response whether it was correct or incorrect. The rules for sorting the cards unexpectedly changed throughout the test after participants made 10 correct responses (Han et al., 2016). Participants were required to flexibly respond to this feedback by shifting to a new rule. The number of switch responses (or categories completed) on the WCST (Berg, 1948, Faustino et al., 2022), i.e., the number of successful rule shifts, was used to assess cognitive flexibility in the present study. The z-standardized switch response score was used for subsequent analyses.

### 2.4 Demographic measures

Parents were asked to report their education level and their children’s age, gender, and grade at baseline and follow-up. Specifically, parental education levels were determined by combining maternal and paternal education, which is commonly used in studies of typically developing children and adolescents (Ganzach, 2000). The twelve response choices ranged from 1 = uneducated to 12 = postgraduate education or above. Higher scores indicate a higher level of education.

### 2.5 Imaging acquisition

All magnetic resonance imaging (MRI) scans were acquired on the same 3 T Siemens Prisma 64-channel head coil at Peking University and Beijing HuiLongGuan Hospital using the same imaging sequences. Prior to scanning, all the children had a mock scanning session using a decommissioned MRI scanner and head coil to acclimate them to the MRI environment. Mock scanning was accompanied by acoustic recordings of the noise produced by gradient coils for each scanning pulse sequence. To further minimize motion, the child’s head was stabilized in the head coil using one foam pad over each ear.

#### 2.5.1 Structural MRI

High-resolution anatomical MRI scans were acquired with an MPRAGE sequence with the following parameters: repetition time (TR) = 2530 ms, echo time (TE) = 2.98 ms, flip angle = 7°, field of view (FOV) = 256 mm × 224 mm, in-plane resolution = 1 mm × 1 mm, sagittal slices = 192, slice thickness = 1 mm and total scan time = 5 min and 58 s

#### 2.5.2 Resting-state fMRI

Blood oxygen level-dependent (BOLD) fMRI was acquired using a whole-brain, single-shot, multislice, echo-planar imaging (EPI) sequence of 240 volumes with the following parameters: repetition time/echo time (TR/TE) = 2000/30 ms, flip angle = 90°, field of view (FOV) = 224 × 224 mm, matrix = 64 × 64, slice thickness = 3.5 mm, slices = 33 and total scan time = 8 min and 6 s. The resulting nominal voxel size was 3.5 × 3.5 × 3.5 mm. During the resting-state scan, subjects were instructed to remain still and awake with their eyes on the fixation cross.

#### 2.5.3 Quality control of MRI data

All MRI scan quality control procedures are described below. a) Individual images were subjected to a careful visual examination by an experienced radiologist to exclude incidental abnormalities, including arachnoid cysts, neuroepithelial cysts, and other intracranial space-occupying lesions. b) Careful visual inspections with a scan rating procedure were separately conducted by five experienced raters using a protocol similar to that used in the Human Connectome Project (Marcus et al., 2013). c) Images considered to have better than fair quality by all the raters were retained. We quantified the head motion of resting-state fMRI as framewise displacement (FD) (Power et al., 2012). Data from participants were excluded if the mean FD exceeded 0.25 mm during resting-state scans (Jenkinson et al., 2002, Xia et al., 2018).

### 2.6 Image processing

The Spatially Unbiased Infratentorial Template (SUIT) toolbox was used for cerebellar voxel-based morphometry (VBM) (version 3.0, https://www.diedrichsenlab.org/imaging/suit.htm) (Diedrichsen et al., 2009). For each subject, the “isolate” function of the toolbox was used to create a mask of the cerebellum and generate gray and white matter segmentation maps. The masked segmentation maps were then normalized to the toolbox template with nonlinear DARTEL normalization. The resulting cerebellar gray matter image was resliced into the SUIT atlas space and smoothed with an 8 mm FWHM isotropic Gaussian kernel, which was small to preserve precision in the definition of cerebellar structures, in line with a previous study (Romer et al., 2018).

Resting state fMRI data preprocessing was performed using fMRIPrep 1.2.3 (Esteban et al., 2019), which is based on Nipype 1.1.6 (Gorgolewski et al., 2011). Preprocessing included the following steps: 1) skull stripping; (2) alignment to the T1w reference; (3) estimation of head motion; 4) slice-time correction; 5) spatial normalization; 6) regressing out the whole brain and white matter signals and twenty-four motion parameters; 7) spatial smoothing with an 8-mm 3D full-width half-maximum kernel; and 8) temporal bandpass filtering (0.01–0.1 Hz).

### 2.7 Statistical analyses

**Fig. 1:**
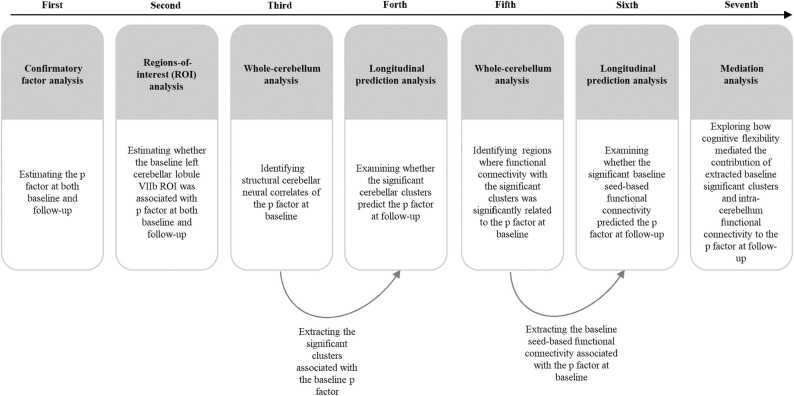
Flowchart of statistical analyses.

All the analyses consisted of seven primary steps (see Fig. 1). First, confirmatory factor analysis (CFA) was used to compute p factor scores of psychiatric problems; CFA is widely used in children and adolescents (Durham et al., 2021, Neumann et al., 2020, Sripada et al., 2021). Briefly, the bifactor model estimates the p factor, representing the overall loadings of psychiatric problems while controlling for the presence of specific dimensions. The p factor score is extracted using the standard regression method from the bifactor model, with a higher p factor score indicating a greater propensity to experience all forms of psychiatric problems. Similar to previous studies, to produce stable factor scores, analyses included all subjects for whom baseline and follow-up SDQ data were available (*N*_baseline_ = 1639, *N*_follow-up_ = 948), rather than only the subsample who completed MRI scans (Moberget et al., 2019, Shanmugan et al., 2016, Vanes et al., 2020) (see Statistical analyses section in the Supplemental Material). Second, we performed a region-of-interest (ROI) analysis to examine the roles of gray matter volumes in the left cerebellar lobule VIIb ROI at baseline in determining the p factor at baseline and follow-up using T1 MRI data at baseline and the p factor data at both baseline and follow-up. Specifically, we extracted the left cerebellar lobule VIIb ROI for each participant from SUIT maps. Subsequently, we examined these associations mentioned above using used a general linear model in which age, gender, site, parental education level, total intracranial volume (TIV), and the autoregressive p factor were the control variables. Third, we conducted whole-cerebellum exploratory analysis of gray matter volume using VBM in SPM12 (http://www.fil.ion.ucl.ac.uk/spm) to identify structural cerebellar neural correlates of the p factor at baseline. Specifically, we used T1 MRI data at baseline and the p factor data at baseline for this analysis. A general linear model was employed in which age, gender, TIV, site, and parental education were the control variables. The significance threshold was set at a voxel-size value of *p* < 0.001 and a familywise error-corrected cluster probability of *p* < 0.001. Fourth, we extracted data from the significant clusters associated with the baseline p factor for each participant, using T1 MRI data at baseline and the p factor data at both baseline and follow-up. This was done to further examine whether the significant cerebellar clusters associated with the baseline p factor predicted the p factor at follow-up. A general linear model was utilized for this analysis in which age, gender, site, parental education level, TIV, and the autoregressive p factor. Fifth, we employed a general linear model to identify regions where functional connectivity with the significant clusters was signiﬁcantly related to the baseline p factor. In this step of the analysis, age, gender, TIV, site, and parental education level were the control variables. Specifically, we used the mean value of the significant clusters as a seed region for the cerebellar functional connectivity analysis using resting-state fMRI data at baseline and the p factor data at baseline. Pearson’s correlation coefficients were calculated to evaluate the correlation between seed-based connectivity and the p factor with DPABI software (http://rfmri.org). The significance threshold was set at a voxel-size value of *p* < 0.001 and a familywise error-corrected cluster probability of *p* < 0.05 (Albaugh et al., 2017, Wang et al., 2023). Sixth, we extracted the baseline seed-based functional connectivity associated with the baseline p factor and conducted a general linear model to examine whether it predicted the p factor at follow-up using resting-state fMRI data at baseline, along with the p factor data at both baseline and follow-up. In this step of the analysis, we controlled for age, gender, TIV, site, parental education level, and the autoregressive p factor. Finally, a series of mediation models were conducted to explore the mediating role of cognitive flexibility in the contribution of the extracted significant baseline clusters and intra-cerebellar functional connectivity to the p factor at follow-up. This analysis utilized data from T1 MRI at baseline, resting-state fMRI at baseline, cognitive flexibility at baseline, and the p factor at both baseline and follow-up. In this step of the analysis, age, gender, site, parental education level, TIV, and the autoregressive p factor were controlled as covariates. The MPLUS software package, version 7.4 (Muthén & Muthén, 1998–2012), was used for the CFA, general linear and mediation models.

## 3 Results

### 3.1 Sample characteristics

All sample characteristics are presented in Table 1 and Supplementary Tables S1–S3. There was no significant difference between the p factor at baseline and follow-up (Table 1). The p factor at follow-up was not significantly correlated with gender, age, or site. However, a higher p factor was correlated with a lower parental education level (Supplementary Tables S2–S3). Worse cognitive flexibility was correlated with a higher p factor. Cognitive flexibility increased with age (Supplementary Tables S2–S3). In the subsequent analysis, parental education level and age were controlled as covariates.

**Table 1:** Characteristics of the popuation in structure MRI study and resting-state fMRI study at baseline and follow-up.

	Structure MRI Study			Resting-state fMRI Study		
	Baseline (*n* = 253)	Follow-up (*n* = 253)	t Value	Baseline (*n* = 230)	Follow-up (*n* = 230)	t Value
Age (*M* ± *SD*)	9.129 ± 1.487	10.145 ± 1.521		9.152 ± 1.464	10.176 ± 1.492	
Gender, Girl, *n* (%)	122 (48.20%)			111 (48.30%)		
Site, 1/ 2	56/197			43/187		
Parental Education Level (*M* ± *SD*)	8.455 ± 2.758			8.530 ± 2.782		
P factor (*M* ± *SD*)	0.011 ± 0.320	0.028 ± 0.349	1.363 (*p* = 0.388)	0.002 ± 0.318	0.031 ± 0.372	1.363 (*p* = 0.174)
Cognitive Flexibility (*M* ± *SD*)	5.60 ± 1.888			5.48 ± 1.772		

**Note.** Site: 1 = Beijing HuiLongGuan Hospital; 2 = Peking University. The parental education levels are determined by combining maternal and paternal education level. Parental education level: 1 = Uneducated; 2 = Primary education; 3 = Junior school; 4 = High school; 5 = Secondary vocational school; 6 = Polytechnic school; 7 = Higher vocational education; 8 = Junior college (parttime); 9 = Junior college (full-time); 10 = Bachelor degree (part-time); 11 = Bachelor degree (full-time); and 12 = Postgraduate education or above.

### 3.2 The risk for general psychopathology (the p factor)

The results indicated that the bifactor model of psychopathology, which consisted of the p factor accounting for shared variance across all psychopathologies and two specific factors accounting for shared variance unique to specific internalizing and externalizing problems, fit the data well at both baseline and follow-up (Supplementary Tables S4–S5). Importantly, the bifactor model of psychopathology was scalar across time, warranting the subsequent analysis of the comparable latent construct of psychopathology (Supplementary Table S6). A higher p factor extracted from the bifactor model indicated a greater propensity to experience all forms of psychopathology. For more detailed information, please see the results section of the Supplemental Material.

### 3.3 Smaller gray matter volume in the left cerebellar lobule VIIb was associated with a higher p factor

The results of ROI analysis showed that smaller gray matter volume in the left cerebellar lobule VIIb ROI at baseline was associated with a higher p factor at baseline (*β* = −0.174, *p* < 0.01, 95% CI [−0.307, −0.050]) and at follow-up after controlling for gender, age, site, parental education level, TIV, and the autoregressive p factor (*β* = −0.169, *p* < 0.005, 95% CI [−0.260, −0.063]). Further analysis revealed that age (*t* = −1.069, *p* = 0.286), gender (*t* = 0.928, *p* = 0.354), site (*t* = −0.077, *p* = 0.939), and parental education level (*t* = 1.598, *p* = 0.111) did not significantly moderate the longitudinal prediction of the left cerebellar lobule VIIb ROI on the follow-up p factor.

**Fig. 2:**
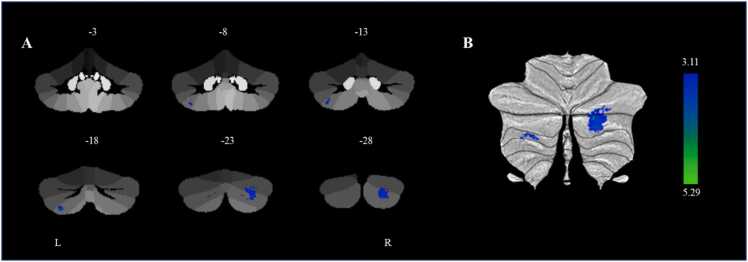
Significant cerebellum clusters of gray matter volume associated with p factor scores. **(**A) A statistical parametric map from the Spatially Unbiased Atlas Template (SUIT) cerebellar-specific, voxel-based morphometry analysis illustrating voxels exhibiting a significant negative correlation with p factor scores within the left cerebellar lobule VIIb and right cerebellar lobule Crus II controlling for gender, age, site, parental education level and total intracranial volume (TIV). (B) Cerebellar regions negatively associated with p factor scores are displayed on a flatmap representation of the cerebellar cortex. The color bar reflects t-scores. L = left; R = right.

Additionally, we conducted a whole-cerebellum exploratory analysis and found that smaller gray matter volumes in the right cerebellar lobule Crus II (cluster 1) (*β* = −0.223, *p* < 0.001, 95% CI [−0.337, −0.109]) and left cerebellar lobule VIIb (cluster 2) (*β* = −0.262, *p* < 0.001, 95% CI [−0.392, −0.132]) at baseline were associated with a higher p factor at baseline controlling for gender, age, site, parental education level, and TIV (Table 2 and Fig. 2). We further found that smaller gray matter volume in the left cerebellar lobule VIIb at baseline was associated with higher p factor at follow-up after controlling for the autoregressive p factor (*β* = −0.211, *p* < 0.005, 95% CI [−0.343, −0.054]). However, the gray matter volume in the right cerebellar lobule Crus II at baseline was not significantly associated with the p factor at follow-up. Therefore, further mediation analysis was not performed for the gray matter volume in the right cerebellar lobule Crus II. Further analysis showed that age (*t* = −0.574, *p* = 0.566), gender (*t* = 0.235, *p* = 0.814), site (*t* = 0.662, *p* = 0.508), and parental education level (*t* = −1.373, *p* = 0.171) did not significantly moderate the longitudinal prediction of the baseline left cerebellar lobule VIIb on the follow-up p factor. These results aligned with the findings from the ROI analysis. We also conducted a linear mixed model analysis, which yielded similar results to the present findings. For more detailed information, please see the results section of the Supplemental Material.

**Table 2:** Differences in cerebellar gray matter volume and with p factor from cerebellar voxel-based morphometry.

Cluster size (k)	Peak Region	MNI Coordinates			T-score	R^2^ (p factor)
		x	y	z		
973	The Right Cerebellar Lobule Crus II	27	-83	-36	5.29	0.031
183	The Left Cerebellar Lobule VIIb	-31	-70	-55	4.31	0.033

**Note.***n* = 473; MNI, Montreal Neurological Institute.

### 3.4 Increased connectivity between the left cerebellar lobule VIIb and the left cerebellar lobule VI was associated with a higher p factor

**Fig. 3:**
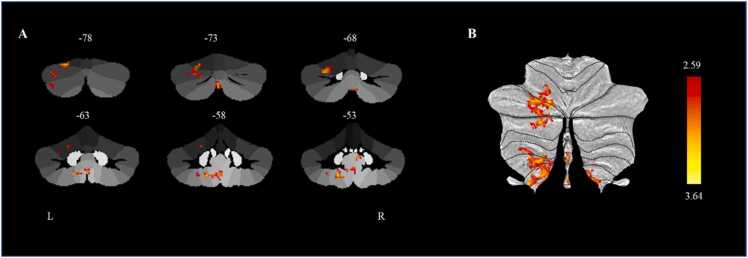
Significant cerebellar intrinsic functional connectivity with the left cerebellar lobule VIIb was associated with p factor scores. (A) A statistical parametric map from the SUIT cerebellar-specific, cerebellar resting functional connectivity analysis illustrating voxels exhibiting a significant positive correlation with p factor scores after controlling for gender, age, site, parental education level and TIV. (B) Cerebellar regions positively associated with p factor scores are displayed on a flatmap representation of the cerebellar cortex. The color bar reflects t-scores. L = left; R = right.

The results showed that a higher p factor at baseline was significantly associated with baseline increased connectivity between the left cerebellar lobule VIIb and several other regions, including left lobules VI (62 voxels), Crus I (103 voxels), Crus II (58 voxels), VIIIb (39 voxels) and IX (60 voxels) at baseline. Among these regions, the peak region was the left cerebellar lobule VI (MNI coordinate: x = −20, y = −78, z = −22), which belongs to the frontoparietal network (Fig. 3). The increased connectivity between the left cerebellar lobule VIIb and left cerebellar lobule VI at baseline predicted higher p factor at follow-up (*β* = 0.140, *p* < 0.05, 95% CI [0.028, 0.251]) after controlling for gender, age, site, parental education level, TIV, and the autoregressive p factor. The linear mixed model analysis also yielded consistent results with the present findings (For detailed information, see the Supplemental Results) Further analysis revealed that age (*t* = −0.054, *p* = 0.338), gender (*t* = −0.176, *p* = 0.119), site (*t* = 0.176, *p* = 0.170), and parental education level (*t* = 0.042, *p* = 0.485) did not significantly moderate the longitudinal prediction of connectivity between the left cerebellar lobule VIIb and left cerebellar lobule VI on the follow-up p factor.

### 3.5 Mediation analysis: cognitive flexibility mediated the gray matter volumes in the left cerebellar lobule VIIb at baseline and its connectivity to the left cerebellar lobule VI at baseline in the prediction of the p factor at follow-up

Worse cognitive flexibility at baseline was associated with smaller gray matter volume in the left cerebellar lobule VIIb at baseline (*β* = 0.219, *p* < 0.001, 95% CI [0.099, 0.345]), increased connectivity between the left cerebellar lobule VIIb and the left cerebellar lobule VI at baseline (*β* = −0.236, *p* < 0.001, 95% CI [−0.346, −0.104]) and higher p factor at follow-up (*β*_Structural MRI Study_ = −0.208, *p* < 0.005, 95% CI [−0.304, −0.08]), *β*_Resting-state fMRI Study_ = −0.247, *p* < 0.001, 95% CI [− 0.387, − 0.125]). Therefore, cognitive flexibility may mediate the contribution of gray matter volume in the left cerebellar lobule VIIb and its connectivity to the left cerebellar lobule VI to the follow-up p factor.

**Fig. 4:**
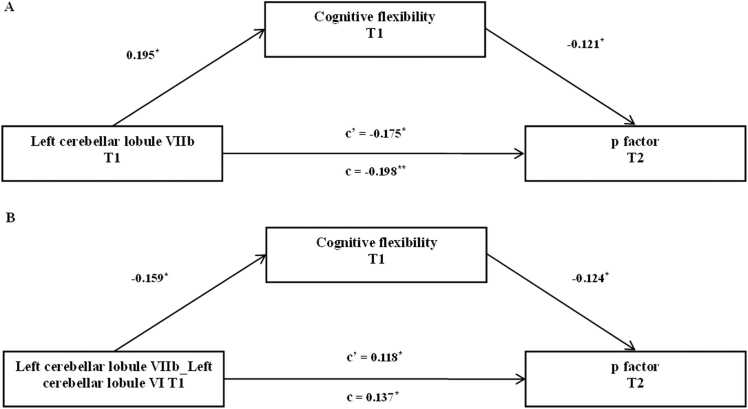
The associations of the left cerebellar lobule VIIb, connectivity between the left cerebellar lobule VIIb and the left cerebellar lobule VI, the p factor and cognitive flexibility. Mediation model using gray matter volume in the left cerebellar lobule VIIb at baseline and connectivity between the left cerebellar lobule VIIb and the left cerebellar lobule VI at baseline as the predictor, cognitive flexibility at baseline as the mediator, and the p factor at the follow-up as the dependent variable. Mediation results are shown as unstandardized regression coefficients. The significance of the indirect effect was assessed using bootstrapped confidence intervals. (A) The smaller volume of the left cerebellar lobule VIIb predicts a higher p factor at follow-up by worse cognitive flexibility. (B) The increased connectivity between left cerebellar lobule VIIb and left cerebellar lobule VI predicts a higher p factor at follow-up by worse cognitive flexibility. Age, gender, site, parental education level, and TIV were used as covariates in the mediation analysis. T1 = baseline, T2 = follow-up. * *p* < 0.05, ^**^p < 0.01^.^

The mediation models revealed that worse cognitive flexibility significantly mediated the relationship between smaller gray matter volume in the left cerebellar lobule VIIb at baseline and a higher p factor at follow-up (11.869% of the total effect size, *β* = −0.0235, *p* < 0.05, 95% CI [−0.0686, −0.0024]) after controlling for gender, age, site, parental education level, TIV, and the autoregressive p factor (Fig. 4A). Similarly, we found that worse cognitive flexibility significantly mediated the link between increased connectivity between the left cerebellar lobule VIIb and the left cerebellar lobule VI at baseline and a higher p factor at follow-up (14.391% of the total effect size, *β* = 0.0197, *p* < 0.05, 95% CI [0.0013, 0.0568]) after controlling for gender, age, site, parental education level, TIV, and the auto regressor of p factor (Fig. 4B). Subsequent analysis showed that age (*t* = −0.648, *p* = 0.518), gender (*t* = .283, *p* = 0.778), site (*t* = 0.646, *p* = 0.519) and parental education level (*t* = −0.340, *p* = 0.734) did not significantly moderate the longitudinal predictive association between the left cerebellar lobule VIIb and the follow-up p factor. Similarly, age (*t* = −0.944, *p* = 0.346), gender (*t* = −1.127, *p* = 0.261), site (*t* = 1.029, *p* = 0.306) and parental education level (*t* = 1.034, *p* = 0.302) did not significantly moderate the longitudinal predictive association between connectivity of the left cerebellar lobule VIIb and the left cerebellar lobule VI and the follow-up p factor.

## 4 Discussion

This study empirically examined the longitudinal predictive associations of cerebellar structure and function with the risk for general psychopathology and the mediating role of cognitive flexibility. We found that a smaller gray matter volume in the left cerebellar lobule VIIb and its increased connectivity to the left cerebellar lobule VI significantly predicted a higher risk for general psychopathology one year later, even after controlling for gender, age, parental education level, TIV and the baseline risk for general psychopathology; moreover, worse cognitive flexibility partially mediated this prediction. This study highlighted the unique contribution of left cerebellar lobule VIIb and its connectivity to left cerebellar lobule VI to the development of the risk for general psychopathology.

Using the longitudinal data for the first time, we found that smaller gray matter volume in the left cerebellar lobule VIIb prospectively predicted a higher risk for general psychopathology regardless of gender, age, and parental education level, which was consistent with previous findings from cross-sectional studies of children and adolescents (Moberget et al., 2019, Romer et al., 2018). Furthermore, previous studies found no significant association between the gray matter volume of the left cerebellar lobule VIIb and the risk for general psychopathology in adults (Romer et al., 2021, Romer and Pizzagalli, 2022). Combined with the longitudinal evidence from this study and the cross-sectional evidence from previous studies, the left cerebellar lobule VIIb may affect the risk for general psychopathology from early childhood to adolescence, which may reflect the still-active development of the cerebellum during this period (Diamond, 2000, Taki et al., 2013, Tiemeier et al., 2010). Additionally, this study provides direct evidence that the neural correlates of psychiatric problems precede the development of those problems in children and adolescents (Albaugh et al., 2019, Shaw et al., 2006, Whelan et al., 2014). Overall, the left cerebellar lobule VIIb may serve as a reliable and unique neural imaging marker for the risk for general psychopathology in school-age children, suggesting a potential path to developing targeted interventions for psychiatric problems in school-age children (McGorry et al., 2006).

This study found for the first time that the intra-cerebellar functional connectivity of the left cerebellar lobule VIIb and the left cerebellar lobule VI prospectively predicted higher risk for general psychopathology in school-age children, regardless of gender, age, or parental education level. The left cerebellar lobule VI belongs to the frontoparietal network (Buckner et al., 2011). The frontoparietal network may be associated with the risk for general psychopathology. Previous studies found that disrupted frontoparietal network connectivity has been reported to be linked to various psychiatric problems, including schizophrenia (Barch and Ceaser, 2012), depression (Kaiser et al., 2015), and bipolar disorder (Anticevic et al., 2014). Recent studies have also shown that the intrinsic functional connectivity of the frontoparietal network is positively correlated with the risk for general psychopathology in young adults (Elliott et al., 2018) and children (Lees et al., 2021). The findings of the present study confirm the importance of the frontoparietal network for the development of the risk for general psychopathology. Moreover, an emerging theory also suggests that the relative integrity of the frontoparietal network is fundamental for managing psychopathology (Cole et al., 2014). Thus, the intra-cerebellar functional connectivity of the left cerebellar lobule VIIb and the left cerebellar lobule VI might be a reliable neural imaging marker for the risk for general psychopathology, which further suggests that its dysfunction may manifest as psychopathology.

We found that cognitive flexibility mediated the longitudinal associations between the left cerebellar lobule VIIb, its connectivity to the left cerebellar lobule VI and the risk for general psychopathology regardless of gender, age, and parental education level, indicating that deficits in cognitive flexibility may underlie the role of cerebellum development in determining the risk for general psychopathology. To our knowledge, this study provides the first empirical evidence of the mediating role of cognitive flexibility in the longitudinal predictive association of cerebellar development with the risk for general psychopathology. On the one hand, the left cerebellar lobule VIIb and its connectivity to the left cerebellar lobule VI may be uniquely associated with cognitive flexibility rather than other executive function components (e.g., working memory) (Moore et al., 2017). According to the dysmetria of thought (DoT) theory (Schmahmann, 1991, Schmahmann et al., 2019), damage to the cerebellum posterior lobes leads to cerebellar cognitive affective syndrome (CCAS)/Schmahmann syndrome, which can involve impairments in cognitive flexibility (Schmahmann, 2004). On the other hand, deficits in cognitive flexibility were found to be an important predictor of the risk for general psychopathology in children and adolescents, which was consistent with the findings of previous studies (Bloemen et al., 2018, Caspi et al., 2014, Zhao et al., 2023). Children with cognitive flexibility deficits may be unable to interrupt biased or ineffective thoughts and actions to pursue new goals and follow new requirements, resulting in difficulty with new situations and the development of maladaptive behaviors (Zelazo, 2020). The present study is a critical step in gaining a better understanding of the risk for general psychopathology. Together with findings from previous studies, the mediating role of cognitive flexibility may be specific to the longitudinal associations between the left cerebellar lobule VIIb, its connectivity within the cerebellum, and the risk for general psychopathology. These findings suggest that early intervention and prevention of psychopathology may target cognitive flexibility, which is supported by the left cerebellar lobule VIIb and its increased connectivity within the cerebellum.

Several limitations should be noted and addressed in future research. First, we followed the participants for a year, and only collected one neural and two behavioral measures, which is insufficient to fully examine the association of the cerebellar developmental trajectory with the risk for general psychopathology. With the continuation of this longitudinal study, we can further address this important research question with three or more data points over a longer period. Second, this study showed that the left cerebellar lobule VIIb and its connectivity to the left cerebellar lobule VI robustly predicted the risk for general psychopathology via cognitive flexibility regardless of gender, age and parental education level. Future research may further examine whether and how other important factors, such as early life adversity and puberty, may influence the longitudinal prediction of the risk for general psychopathology. Third, this study identified a unique contribution of cerebellar structural and functional connectivity abnormalities. Since the functions of the cortex and cerebellum may affect each other during development, future research should integrate analysis of the cortex and cerebellum to broaden and deepen our understanding of the interaction between the brain and psychiatric problems.

In conclusion, cerebellar structural and functional connectivity abnormalities may precede the risk for general psychopathology in childhood and adolescence, partially via cognitive flexibility. Reduced gray matter volume in the left cerebellar lobule VIIb and the increased connectivity of this region to the left cerebellar lobule VI may serve as neural imaging markers for the risk for general psychopathology. Cognitive flexibility can be considered a target for preventing and intervening in general mental health problems in children and early adolescents.

## Funding

The work was supported by the STI 2030—Major Projects (2021ZD0200503) and the 10.13039/501100013314111 Project (BP0719032).

## Declaration of Competing Interest

The authors declare that there are no conflicts of interest.

## Data Availability

The raw data supporting the conclusions of this article were from the Children School Functions and Brain Development Project (CBD, Beijing Cohort), which will be soon made public.
