# Supplementary material for: Reduced volume of the left cerebellar lobule VIIb and its increased connectivity within the cerebellum predict more general psychopathology one year later via worse cognitive flexibility in children
Source: Dev Cogn Neurosci. 2023 Sep 7;63:101296. doi: 10.1016/j.dcn.2023.101296 (PMC10507200; doi:10.1016/j.dcn.2023.101296)
Supplement: Supplementary file 1 — Supplementary material [file mmc1.docx]

**Reduced volume of the left cerebellar lobule VIIb and** **its increased connectivity** **within the cerebellum predict more general psychopathology one year later via worse cognitive flexibility in children**

**Supplemental Material**

# Methods

## *Statistical analyses*

We conducted confirmatory factor analysis (CFA) to examine three competing latent factor models at baseline and follow-up: (a) a one-factor model that all the items loaded only onto a single factor of psychopathology; (b) a two-correlated-factor model that represented the independent and correlated internalizing and externalizing problems; and (c) a bifactor model that introduced a general psychopathology factor (p factor), on which every observed variable loaded, in addition to loading onto the independent internalizing or externalizing problems. The weighted least-square means and variances (WLSMV) estimator is the most suited, as SDQ was a category and non-normal data (Finney & DiStefano, 2006). And WLSMV estimator also provided consistent estimates when data are missing at random with respect to covariates (Caspi et al., 2014). Once the optimal model was identified, factor scores from this model were used for the subsequent analysis. To produce stable factor scores, analyses included all subjects for whose SDQ data were available (*N*_baseline_ = 1,639, *N*_follow-up_ = 948), rather than only the subsample who completed MRI scans (Moberget et al., 2019; Shanmugan et al., 2016; Vanes et al., 2020).

# Results

## *The latent structure of* *psychopathology*

We tested three competing latent factor models with CFA. Table S4 shows that at baseline, the two-factor model performed significantly better than the one-factor model (Δχ^2^ = 224.90, *p* < 0.0001), and the bifactor model performed significantly better than the two-factor model (Δχ^2^ = 364.45, *p* < 0.0001). Therefore, the bifactor model exhibited a better fit than other models at baseline, indicating that the p factor together with specific internalizing and externalizing problems may better describe psychopathology. For the p factor that captures the shared variances among multiple forms of psychopathology, the factor loadings from the observed variables were positive and significant (range: 0.19~0.68, all *ps* < 0.05), except the item 7 (“Generally obedient, usually does what adults request”: 0.048, *p* > 0.05) (see Table S5). The factor loadings on the p factor from the observed variables about emotional symptoms and peer relationship were stronger than the corresponding loadings on the latent internalizing problems (*p* < 0.01), suggesting that the emotional symptoms and peer relationship may indicate the p factor rather than being specific to the internalizing problems. For the latent internalizing problems unique to various internalizing problems, the factor loadings were significant (range: -0.45~0.48, all *ps* < 0.05), except item 23 (“Gets on better with adults than with other children”: 0.072, *p* > 0.05) (see Table S5). In contrast, the factor loadings from the observed variables about the conduct disorders and hyperactive symptoms on the p factor were similar to that of corresponding variables on the externalizing problems (*p* = 0.91), suggesting that the conduct disorders and hyperactivity may be a combination of the externalizing problems and the general tendency to psychopathology. The factor loadings were positive and significant for the latent externalizing factor unique to various externalizing problems (range: 0.13~0.70, all *ps* < 0.05) (see Table S5).

Similarly, at one-year-follow-up, the bifactor model exhibited a better fit than the other two models (see Table S4), indicating again that the p factor together with specific internalizing and externalizing problems may better describe psychopathology. The factor loadings on the p factor and externalizing factor were positive and significant (the p factor: range: 0.127~0.583; externalizing problems: range: 0.15~0.72; all *ps* < 0.05). The factor loadings on internalizing problems were significant (range: -0.53~0.53, all *ps* < 0.05), except item 6 (“Rather solitary, tends to play alone”: 0.059, *p* > 0.05) and item 19 (“Picked on or bullied by other children”: 0.059, *p* > 0.05) (see Table S5).

In addition to the configural invariance across time shown above, we further examined the metric and scaler invariance of the latent construct of psychopathology using longitudinal invariance analysis. The bifactor latent construct of psychopathology at scalar invariance was established over time (see Table S6). The ΔCFI and ΔRMSEA were below 0.01, indicating that scaler invariance across time was demonstrated. Therefore, the bifactor models were scalar invariant across time, warranting the subsequent analysis of the comparable latent construct of psychopathology at both baseline and follow-up.

## *Supplemental results*

We also conducted linear mixed models where site and gender were random variables, and found that smaller gray matter volumes in the left cerebellar lobule VIIb at baseline (*β* = -0.169, *p* < 0.05, 95% CI [-0.313, -0.024]) and the increased connectivity between the left cerebellar lobule VIIb and left cerebellar lobule VI at baseline (*β* = 0.137, *p* < 0.05, 95% CI [0.028, 0.246]) predicted higher p factor at follow-up after controlling for age, parental education level, TIV, and the auto regressor of p factor.

Moreover, to assess the unique contribution of the gray matter volume in the left cerebellar lobule VIIb to the development of the p factor, we performed stepwise regression analysis. We found that smaller gray matter volumes in the left cerebellar lobule VIIb (cluster 1) (*β* = -0.251, *p* < 0.01, 95% CI [-0.430, -0.073]) and the left cerebellar lobule VIIb ROI (*β* = -0.201, *p* < 0.05, 95% CI [-0.425, -0.023]) at baseline were associated with a higher p factor at follow-up, controlling for gender, age, site, parental education level, TIV and gray matter volume of the right cerebellar Crus II.

**Supplemental Tables**

**Table S1**

Characteristics of the population in structure MRI study and resting-state fMRI study at baseline

|  | Baseline | |
| --- | --- | --- |
|  | Structural MRI study (*n* = 473) | Resting-state fMRI study (*n* = 439) |
| Age (*M* ± *SD*) | 9.177 ± 1.465 | 9.220 ± 1.470 |
| Gender, Girl, *n* (%) | 211 (44.60%) | 199 (45.30%) |
| Site, Beijing HuiLongGuan Hospital/ Peking University | 107/366 | 93/336 |
| Parental Education Level (*M* ± *SD*) | 8.410 ± 2.754 | 8.394 ± 2.777 |
| p factor (*M* ± *SD*) | 0.0096 ± 0.316 | 0.0049 ± 0.314 |
| Cognitive Flexibility (*M* ± *SD*) | 5.570 ± 1.839 | 5.590 ± 1.821 |
| Total Intracranial volume (*M* ± *SD*) | 1561.079 ± 131.427 | 1561.225 ± 131.479 |

**Table S2**

The mean, standard deviation and correlations between main variables in structure MRI study

| Variables | 1 | 2 | 3 | 4 | 5 | 6 | 7 | 8 |
| --- | --- | --- | --- | --- | --- | --- | --- | --- |
| 1. Gender^ | --- |  |  |  |  |  |  |  |
| 2. Site^ | 0.000 | --- |  |  |  |  |  |  |
| 3. Age T1 | 0.064 | 0.128^*^ | --- |  |  |  |  |  |
| 4. Age T2 | 0.070 | 0.124^*^ | 0.990^***^ | --- |  |  |  |  |
| 5.Parental Education Level | -0.034 | -0.020 | -0.083 | -0.071 | --- |  |  |  |
| 6. p Factor T1 | -0.080 | 0.028 | 0.004 | -0.003 | -0.169^**^ | --- |  |  |
| 7. p Factor T2 | -0.018 | 0.063 | 0.015 | 0.012 | -0.167^**^ | 0.594^***^ | --- |  |
| 8. Cognitive Flexibility T1 | -0.012 | 0.022 | 0.141^**^ | 0.151^**^ | 0.155^**^ | -0.245^***^ | -0.208^**^ | --- |

**Note.** T1= baseline; T2 = follow-up; ^ Spearman correlation; ^*^*p* < 0.05; ^**^*p* < 0.01; ^***^*p* < 0.001.

**Table S3**

The mean, standard deviation and correlations between main variables in resting-state fMRI study

| Variables | 1 | 2 | 3 | 4 | 5 | 6 | 7 | 8 |
| --- | --- | --- | --- | --- | --- | --- | --- | --- |
| 1. Gender^ | --- |  |  |  |  |  |  |  |
| 2. Site^ | 0.006 | --- |  |  |  |  |  |  |
| 3. Age T1 | 0.126 | 0.152^*^ | --- |  |  |  |  |  |
| 4. Age T2 | 0.136^*^ | 0.146^*^ | 0.989^***^ | --- |  |  |  |  |
| 5.Parental Education Level | -0.040 | -0.069 | -0.108 | -0.089 | --- |  |  |  |
| 6. p Factor T1 | -0.078 | 0.025 | 0.022 | 0.021 | -0.118 | --- |  |  |
| 7. p Factor T2 | -0.008 | 0.104 | 0.076 | 0.074 | -0.197^**^ | 0.557^***^ | --- |  |
| 8. Cognitive Flexibility T1 | 0.046 | 0.036 | 0.204^**^ | 0.220^**^ | 0.127 | -0.207^**^ | -0.247^***^ | --- |

**Note.**T1= baseline; T2 = follow-up; ^ Spearman correlation; ^*^*p* < 0.05; ^**^*p* < 0.01; ^***^*p* < 0.001.

| Time point | Model | *n* | χ^2^ | *df* | RMSEA | RMSEA 90%CI | CFI | Model comparison |
| --- | --- | --- | --- | --- | --- | --- | --- | --- |
| T1 | 1: One-factor | 1,639 | 1900.185 | 170 | 0.079 | [0.076-0.082] | 0.798 |  |
|  | 2: Two-factor | 1,639 | 1393.943 | 169 | 0.067 | [0.063-0.070] | 0.857 | Δχ^2^: 2 vs 1(1) = 224.900, *p* < 0.0001 |
|  | 3: Bifactor | 1,639 | 994.374 | 150 | 0.059 | [0.055-0.062] | 0.901 | Δχ^2^: 3 vs 2(19) = 364.450, *p* < 0.0001 |
| T2 | 1: One-factor | 948 | 965.895 | 170 | 0.070 | [0.066-0.075] | 0.823 |  |
|  | 2: Two-factor | 948 | 785.172 | 169 | 0.062 | [0.058-0.066] | 0.863 | Δχ^2^: 2 vs 1(1) = 84.451,  *p* < 0.0001 |
|  | 3: Bifactor | 948 | 564.011 | 150 | 0.054 | [0.049-0.059] | 0.908 | Δχ^2^: 3 vs 2(19) = 213.213, *p* < 0.0001 |

**Table S4**

Model fit statistics from three different confirmatory factor models

**Note.** RMSEA = root mean square error of approximation; CI = confidence interval; CFI = comparative fit index; T1= baseline, T2 = follow-up.

**Table S5**

Standardized factor loadings for bifactor models of psychiatric problems at baseline and follow-up

|  | **Time Point** | | | |
| --- | --- | --- | --- | --- |
|  | T1 | | T2 | |
| Items | S-FL | P-FL | S-FL | P-FL |
| **Internalizing Problems** |  |  |  |  |
| 3. Often complains of headaches | 0.230^***^ | 0.428^***^ | 0.354^***^ | 0.485^***^ |
| 8. Many worries | 0.420^***^ | 0.440^***^ | 0.525^***^ | 0.477^***^ |
| 13. Often unhappy, downhearted | 0.250^***^ | 0.676^***^ | 0.434^***^ | 0.583^***^ |
| 16. Nervous or clingy in new situations | 0.407^***^ | 0.492^***^ | 0.243^***^ | 0.519^***^ |
| 24. Many fears | 0.476^***^ | 0.480^***^ | 0.454^***^ | 0.558^***^ |
| 6. Tends to play alone | 0.108^*^ | 0.367^***^ | 0.059 | 0.480^***^ |
| 11. Least one good friend(R) | -0.327^***^ | 0.300^***^ | -0.277^***^ | 0.417^***^ |
| 14. Generally liked by other children(R) | -0.446^***^ | 0.563^***^ | -0.532^***^ | 0.574^***^ |
| 19. Picked on or bullied | 0.125^**^ | 0.569^***^ | 0.059 | 0.575^***^ |
| 23. Better with adults | 0.072 | 0.188^***^ | 0.167^**^ | 0.127^**^ |
| **Externalizing Problems** |  |  |  |  |
| 5. Often has temper tantrums or hot tempers | 0.127^***^ | 0.535^***^ | 0.171^***^ | 0.545^***^ |
| 7. Generally obedient (R) | 0.231^***^ | 0.048 | 0.145^**^ | 0.207^***^ |
| 12. Often fights with other children | 0.175^**^ | 0.579^***^ | 0.214^*^ | 0.416^***^ |
| 18. Often lies of cheats | 0.289^***^ | 0.507^***^ | 0.349^***^ | 0.518^***^ |
| 22. Steals from home, school, or elsewhere | 0.276^**^ | 0.383^***^ | 0.311^**^ | 0.402^***^ |
| 2. Restless, overactive | 0.581^***^ | 0.437^***^ | 0.616^***^ | 0.383^***^ |
| 10. Constantly fidgeting or squirming | 0.501^***^ | 0.454^***^ | 0.597^***^ | 0.354^***^ |
| 15. Easily distracted, concentrating wanders | 0.670^***^ | 0.489^***^ | 0.719^***^ | 0.469^***^ |
| 21. Thinks things out before acting (R) | 0.581^***^ | 0.321^***^ | 0.313^***^ | 0.564^***^ |
| 25. Sees tasks through to the end (R) | 0.702^***^ | 0.288^***^ | 0.527^***^ | 0.513^***^ |

**Note.** S-FL scale-specific factor loadings; P-FL factor loadings on the p factor. Items followed by (R) are reverse-coded. T1= baseline; T2 = follow-up; ^*^*p* < 0.05; ^**^*p* < 0.01; ^***^*p* < 0.001.

**Table S6**

**Measurement invariance of bifactor models across time**

| Model | χ^2^ | *df* | RMSEA | RMSEA 90%CI | CFI | ΔCFI | ΔRMSEA |
| --- | --- | --- | --- | --- | --- | --- | --- |
| 1: Configural Invariance | 1219.872 | 300 | 0.057 | [0.053-0.060] | 0.907 |  |  |
| 2: Metric Invariance (weak) | 1214.756 | 360 | 0.050 | [0.047-0.053] | 0.914 | 0.007 | -0.007 |
| 3: Scalar Invariance (strong) | 1160.173 | 354 | 0.049 | [0.051-0.057] | 0.918 | 0.004 | -0.001 |

**Note.** RMSEA = root mean square error of approximation; CI = confidence interval; CFI = comparative fit index.

**References**

Caspi, A., Houts, R. M., Belsky, D. W., Goldman-Mellor, S. J., Harrington, H., Israel, S., Meier, M. H., Ramrakha, S., Shalev, I., Poulton, R., & Moffitt, T. E. (2014). The p Factor: One general psychopathology factor in the structure of psychiatric disorders? *Clinical Psychological Science*, *2*(2), 119‒137. <https://doi.org/10.1177/2167702613497473>

Finney, S. J., & DiStefano, C. (2006). Nonnormal and categorical data in structural equation modeling. In G.R. Hancock & R. O. Mueller (Ed.), *Structural equation modeling: A second course* (pp. 269‒314). Greenwich: Information Age Publishing.

Moberget, T., Alnæs, D., Kaufmann, T., Doan, N. T., Córdova-Palomera, A., Norbom, L. B., Rokicki, J., van der Meer, D., Andreassen, O. A., & Westlye, L. T. (2019). Cerebellar gray matter volume is associated with cognitive function and psychopathology in adolescence. *Biological Psychiatry*, *86*(1), 65‒75. <http://dx.doi.org/10.1016/j.biopsych.2019.01.019>

Shanmugan, S., Wolf, D. H., Calkins, M. E., Moore, T. M., Ruparel, K., Hopson, R. D., Vandekar, S. N., Roalf, D. R., Elliott, M. A., Jackson, C., Gennatas, E. D., Leibenluft, E., Pine, D. S., Shinohara, R. T., Hakonarson, H., Gur, R. C., Gur, R. E., & Satterthwaite, T. D. (2016). Common and dissociable mechanisms of executive system dysfunction across psychiatric disorders in youth. *American Journal of Psychiatry*, *173*(5), 517‒526. <http://dx.doi.org/10.1176/appi.ajp.2015.15060725>

Vanes, L. D., Moutoussis, M., Ziegler, G., Goodyer, I. M., Fonagy, P., Jones, P. B., Bullmore, E. T., & Dolan, R. J. (2020). White matter tract myelin maturation and its association with general psychopathology in adolescence and early adulthood. *Human Brain Mapping*, *41*(3), 827‒839. <http://dx.doi.org/10.1002/hbm.24842>
